# Cueing distractors is effective when the incentive to suppress is high

**DOI:** 10.3758/s13414-025-03075-w

**Published:** 2025-05-05

**Authors:** Anna Heuer, Anna Schubö

**Affiliations:** 1 Department of Psychology, Humboldt-Universität zu Berlin, Berlin, Germany; 2 Experimental and Biological Psychology, Philipps-Universität Marburg, Marburg, Germany

**Keywords:** Attention, Selective, Attentional capture

## Abstract

Avoiding distraction is critical for our ability to focus, and recent years have seen an increased interest in attentional suppression mechanisms. We now know that we implicitly learn about statistical regularities of our environment, which facilitates inhibition, but it remains unclear if distractors can also be suppressed voluntarily when advance information about their occurrence becomes available. Reasoning that such top-down suppression is likely an effortful process requiring a certain degree of motivation, we aimed to show that distractor cueing can effectively reduce distraction when the incentive is high. In an additional singleton search task, we maximized the incentive to suppress by presenting cues that validly indicated the distractor’s specific location and colour, and by rewarding successful suppression. For correct responses, participants received either a low or high reward, depending on distractor colour. Responses were faster in trials with predictive cues than in trials with cues that did not provide any information. These cueing benefits increased over the course of the experiment. Reward magnitude also affected reaction times, indicating that high-reward singletons were more distracting, but did not interact with cueing condition. This performance pattern was complemented by modulations of the P_D_, a lateralized event-related potential component reflecting active suppression: Smaller amplitudes, indicating that less suppression was required, were observed for low- versus high-reward distractors and, more importantly, for distractors following predictive versus nonpredictive cues. These findings provide proof-of-principle that salient distractors can be anticipatorily suppressed in a top-down manner and highlight the importance of motivation for this voluntary operation.

**Supplementary Information:**

The online version contains supplementary material available at 10.3758/s13414-025-03075-w.

## Introduction

To navigate our typically rich visual environments, we need to ignore irrelevant information as much as we need to focus on what is important for our current goals. In fact, more than ever, we are surrounded by stimuli that were downright designed to capture our attention and distract us from what we are doing—such as advertisement billboards, video screens, and our smartphones. The neglect of some parts of our surroundings has long been considered a necessary by-product of attending to others, but recent years have seen a surge in research on how information can be actively suppressed and how that is different from merely not attending to something.

We now know that suppression mechanisms are manifold and can be distinguished along different dimensions (e.g., Chelazzi et al., 2019; van Moorselaar & Slagter, 2020), albeit a clear taxonomy has not yet been proposed (but see Luck et al., 2021, for a common framework for models on attentional control). A distinction that appears to be critical with respect to the efficacy of distractor suppression is the distinction between suppression mechanisms depending on previous experience with distracting information on the one hand and mechanisms under top-down control on the other hand. Experience-dependent suppression is driven by factors such as the probability of distractor presence (e.g., Geng et al., 2017; Marini et al., 2013) or statistical regularities of distractor locations (e.g., Failing, Wang et al., 2019b; Ferrante et al., 2018; Sauter et al., 2018; Wang & Theeuwes, 2018b) or surface features (e.g., Failing, Feldmann-Wüstefeld et al., 2019a; Gaspelin & Luck, 2018a; Stilwell et al., 2019; Vatterott & Vecera, 2012). It occurs involuntarily and relies on implicit learning, emerging over time. Top-down suppression, by contrast, is voluntary and explicit. Experimentally, this is typically studied with trial-to-trial cueing of distractor features prior to their presentation. Whereas a large and growing body of evidence has demonstrated that suppression relying on previous experience with distracting stimuli is effective and robust, it remains a matter of debate whether the same applies to top-down suppression (for recent reviews, see Cunningham & Egeth, 2016; Geng et al., 2019; Noonan et al., 2018; van Moorselaar & Slagter, 2020).

Studies investigating cued suppression with negative cues and non-salient distractors have mostly observed performance benefits, indicating that advance knowledge about upcoming distractors on a trial-to-trial basis could be used to facilitate their suppression (e.g., Arita et al., 2012; Carlisle & Nitka, 2019; Chao, 2010; Conci et al., 2019; Munneke et al., 2008; Zhang et al., 2020; but see Beck & Hollingworth, 2015; Becker et al., 2015). For salient distractors such as colour singletons, however, some studies reported behavioural cueing benefits or electrophysiological signatures of successful suppression (e.g., Chang et al., 2018; Heuer & Schubö, 2019; Van Zoest et al., 2021) while others failed to observe an effect of informative cues (Salahub & Emrich, 2021; Wang & Theeuwes, 2018a). Considering this overall mixed evidence, it seems reasonable to assume that anticipatory top-down suppression of distractors is only possible to some degree or under certain circumstances, respectively. Boundary conditions for successful suppression might, for instance, be defined by details of the task and design that influence participants’ motivation to make use of the cues and that differ across studies—including cue validity, cue specificity, overall search difficulty (see also Conci et al., 2019) or the time available to process the cue (see also Moher & Egeth, 2012). This reasoning assumes that trial-to-trial distractor suppression is a cognitively demanding process which, simply put, needs to be worth the effort. A low cue validity (e.g., <50% in Wang & Theeuwes, 2018a) might not provide enough incentive to attempt suppression. A high cue specificity (e.g., cues indicating the exact location of an upcoming salient distractor, as in Chang et al., 2018), conversely, might increase participants’ motivation to make use of the information provided. For the suppression of salient distractors, when the effort required to overcome distraction is arguably larger, such factors that affect the incentive to make use of distractor cues might accordingly be particularly important.

A critical role of the incentive to suppress could also account for pronounced interindividual differences in cueing benefits, for example in our previous work (Heuer & Schubö, 2019). When cueing the upcoming location of a colour singleton distractor, we did not observe a significant performance benefit at the group level. At the individual level, however, there was a considerable number of participants who exhibited cueing benefits (up to ~20 ms), and larger individual cueing benefits were associated with larger differences in the P_D_, an event-related potential (ERP) component of the EEG reflecting active suppression of irrelevant stimuli. It thus seemed that there were some participants who successfully suppressed the salient distractors, and others who did not.

Here, we aimed to test the efficacy of cueing salient distractor features under conditions that should motivate participants to make use of the negative cues. To this end, we employed a powerful incentive to increase participants’ motivation to make use of the distractor cues to facilitate suppression: monetary reward. Reward profoundly shapes the way we perceive and interact with our environment. For the visual domain, it has, for example, been shown to affect the deployment of attention (Anderson, 2016; Chelazzi et al., 2013; Failing & Theeuwes, 2017), the allocation of memory resources (e.g., Gong & Li, 2014; Heuer & Schubö, 2018; Klink et al., 2017; Thomas et al., 2016), and the execution of visually guided movements (Chapman et al., 2015; Moher et al., 2015).

The general prospect of reward is known to increase motivational engagement in a task, acting as an incentive for participants to perform optimally to maximize their monetary gain and has been linked to cognitive and attentional control (e.g., Chelazzi et al., 2013; Engelmann & Pessoa, 2007; Pessoa, 2009; Sanada et al., 2013; Sawaki et al., 2015; Small et al., 2005). In addition to this broad influence on overall performance, associations between specific stimuli and reward also selectively modulate the processing of these motivationally salient stimuli: Visual processing is typically biased towards stimuli or objects that predict some sort of gain, even when this bias conflicts with other current goals. For instance, task-irrelevant distractors that have been learned to signal reward have repeatedly been demonstrated to capture attention and impair target detection (Anderson et al., 2011a, b; Della Libera & Chelazzi, 2009; Failing & Theeuwes, 2015; Feldmann-Wüstefeld et al., 2016; Le Pelley et al., 2015). Such value-driven attentional capture is usually influenced by reward magnitude and stronger for stimuli associated with a high reward as compared with stimuli associated with a low reward. In some studies, the association with reward was established in training tasks prior to the search task, in which reward could no longer be earned, so that impaired performance did not result in a monetary loss (e.g., Anderson et al., 2011a, b; Della Libera & Chelazzi, 2009; Theeuwes & Belopolsky, 2012). But even when reward delivery was contingent on search performance, meaning that any distraction increased the likelihood that no reward would be obtained on a given trial, capture by reward-associated distractors could be observed (e.g., Failing et al., 2015; Le Pelley et al., 2015).

Considering these findings on value-driven capture, one could assume that an association with reward would hamper the voluntary suppression of the respective distractors rather than facilitating it. One study, however, has shown that cueing reward-associated distractors can, at least under certain conditions, improve target selection. Gong et al. (2016) had participants search for a target presented among several distractors, with half of all items presented in one colour and the other half in another. Valid colour cues presented prior to the search display indicated the colour in which the target would not be presented and that could accordingly be ignored. When the cued colour had previously been associated with a high reward, responses were found to be faster as compared with when it was associated with a low reward or a neutral colour. The authors proposed that the enhanced working memory representation (e.g., Gong & Li, 2014; Heuer & Schubö, 2018) of the high-reward item can be used to guide top-down attention and thereby facilitate distractor suppression. It should be noted that the design of this task might have promoted top-down control to begin with—items did not differ in their physical salience and participants were forced to adopt a feature-specific search mode. But these findings show that, in principle, introducing reward to a distractor cueing task might increase the efficacy of the cues by overall increasing participants’ motivation and by strengthening top-down control via enhanced working memory for distractor features.

### Rationale

With this study, we aimed to replicate previous findings of active anticipatory suppression of salient distractors (Chang et al., 2018; Heuer & Schubö, 2019; Van Zoest et al., 2021) and to render the distractor cues behaviourally more effective by increasing the incentive to make use of them. To achieve “optimal inhibition conditions”,^1^ cues (i) were spatially specific and (ii) reliably indicated both the location and the colour of the upcoming distractor (100% validity). Moreover, (iii) participants received a monetary reward for successful distractor suppression (i.e., correct search performance).

In a cued additional singleton search task, participants had to report the orientation of a line embedded in a diamond-shaped target that was presented among several circle-shaped distractors. One of the distractors was a salient colour singleton. Distractor cues, which were either fully predictive or nonpredictive of the location and colour of the singleton distractor, were presented before the search display, leaving ample time for participants to make use of the information they provided. For correct responses, participants received points that were converted into monetary reward at the end of the experiment. Reward magnitude was coupled to the colour of the singleton: One distractor colour was associated with a low and the other with a high reward. As we were primarily interested in the reduction of distraction with predictive cues rather than in the overall attentional capture by the singleton—and to achieve a more parsimonious design—we did not include trials without a singleton; to quantify the reduction of distraction we instead used trials with neutral, nonpredictive cues as baseline. We predicted that reaction times would be shorter with predictive than with nonpredictive cues, and that these cueing benefits would be larger in trials with a high-reward distractor than in trials with a low-reward distractor (see Gong et al., 2016).

As in our previous study (Heuer & Schubö, 2019), we systematically lateralized the presentation of the target and the singleton distractor to isolate ERP components specifically associated with target or distractor processing: Either the target or the singleton distractor was presented laterally, while the other was presented centrally on the vertical midline (Hickey et al., 2009). With this configuration, lateralized ERPs are not affected by the centrally presented item. We were interested in the distractor positivity (P_D_) elicited by lateral distractors, and the N2pc elicited by lateral targets. Both are lateralized ERP components that can be observed at parieto-occipital electrodes in the N2 time range—the P_D_ is a positive deflection that is larger over the hemisphere contralateral to to-be suppressed stimuli relative to the ipsilateral hemisphere, whereas the N2pc is a larger negative deflection contralateral to a target, respectively.

The P_D_ is an established marker of distractor suppression (e.g., Feldmann-Wüstefeld et al., 2020; Gaspelin & Luck, 2018b, c; Gaspelin et al., 2023; Sawaki & Luck, 2010). Its amplitude, for example, increases with increasing levels of suppression (i.e., a larger amplitude is typically taken to reflect that more suppression was applied; e.g., Feldmann-Wüstefeld et al., 2015, 2021; Heuer & Schubö, 2019), is larger in trials with shorter reaction times, indicative of effective suppression (Gaspar & McDonald, 2014; Sawaki et al., 2012), scales with the number of items to be suppressed (Feldmann-Wüstefeld & Vogel, 2018), and is larger in individuals with greater attentional control (Feldmann-Wüstefeld et al., 2018; Gaspar et al., 2016). The N2pc, by contrast, is thought to reflect the selection and enhancement of relevant information. Its amplitude is, for instance, modulated by the number of targets (Munneke et al., 2013) and larger when target processing is facilitated and more efficient (Feldmann-Wüstefeld et al., 2015).

Based on our previous findings, we predicted that the P_D_ would be reduced in trials with predictive cues relative to the P_D_ in trials with nonpredictive cues, indicating that less suppression was required upon distractor presentation when advance information about its location and identity was available beforehand, allowing for anticipatory suppression. If reward facilitates distractor suppression, this P_D_ reduction might be more pronounced for distractors associated with a high reward. The target N2pc, by contrast, should not be modulated by cue condition, confirming that the distractor cues selectively facilitated distractor suppression but did not affect processing of the target.

## Methods

### Participants

Sixty students of Philipps-Universität Marburg participated in the experiment for course credit or monetary compensation. We chose to collect data from a sample larger than the estimated sample size (~40 participants) required to detect small to medium effects, as we observed for the P_D_ in our previous work on distractor cueing (Heuer & Schubö, 2019), with an alpha level of.05 and a power of.80 (Faul et al., 2007) to ensure that enough datasets would remain after EEG artifact rejection. Data from ten participants had to be excluded from the analyses: three because too many trials (>20%) contained ocular or muscle artifacts in the EEG, three because of excessive alpha activity, three because they reported in the postexperimental questionnaire that they had ignored the cues and one because search performance did not exceed chance level. The final sample of 50 participants consisted of 37 women and 13 men (mean age: 22 years; age range: 19–29 years). Participants were naïve to the purpose of the experiment and had normal or corrected-to-normal visual acuity and colour vision, which were tested with the OCULUS Binoptometer 3 (OCULUS Optigkeräte GmbH, Wetzlar, Germany). The experiment was conducted in accordance with the ethical standards laid down in the Declaration of Helsinki and approved by the Ethics Committee of the Faculty of Psychology at the University of Marburg. All participants provided informed written consent.

### Apparatus and stimuli

Participants sat in a dimly lit and electrically shielded room, facing a monitor (22-in., 1,680 × 1,050 pixels) placed at 104 cm from their eyes. A Windows PC using E-Prime 2.0 software (Psychology Software Tools, Inc.) controlled stimulus presentation and response collection. Participants responded by pressing buttons on the back of a gamepad (Microsoft SideWinder USB) with the index finger of their left or right hand.

**Fig. 1:**
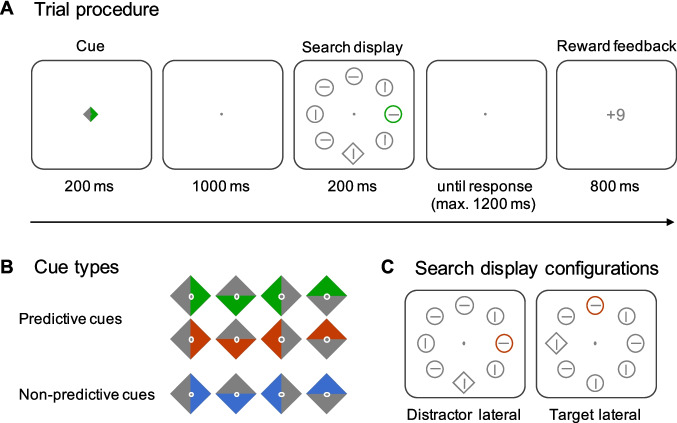
Task and stimuli. **A** Trial procedure. Each trial started with the presentation of a distractor cue: Predictive cues validly indicated the location and colour of the upcoming salient colour singleton, whereas nonpredictive cues provided no such information. After an interval of 1,000 ms, the search display was presented, which contained a diamond-shaped target presented along with seven circle-shaped distractors, one of which was a salient colour singleton (red or green). Participants had to report the orientation (horizontal or vertical) of the line embedded in the target. A response display was present until response or for up to 1,200 ms. At the end of each trial, reward feedback appeared for 800 ms. For correct responses, participants received a low (+1) or a high (+9) reward. Reward magnitude depended on the colour of the singleton, and the reward association was balanced across participants. There was no reward (+0) for incorrect responses. The example shows a trial, in which a predictive cue indicates the location of an upcoming green distractor that is associated with a high reward. **B** Cue types. All cues were diamond-shaped and had one coloured side. Predictive cues validly indicated the location (right, bottom, left, top) and colour (red or green) of the upcoming singleton distractor. Nonpredictive cues were always grey/blue and their orientation was randomly chosen on each trial. **C** Search display configurations. Targets and singleton distractors were arranged in two different configurations: Either the distractor was presented laterally (left or right) and the target centrally (top or bottom), or vice versa. (Colour figure online)

Search displays consisted of seven circle-shaped distractors and one diamond-shaped target, each subtending 1.8 degrees of visual angle (dva; see Fig. 1A and B for examples). These items were presented at eight fixed locations arranged on an imaginary circle (eccentricity of 5.45 dva from fixation) along the vertical, horizontal, and diagonal axes. The distance from the centre of one item to the centre of the next item was 4.3 dva. A horizontally or vertically oriented line with a length of 1.1 dva was embedded in each item. All target and distractor stimuli, including the embedded horizontal and vertical lines, were grey (RGB: 134|134|134), except for the singleton distractor, which was either red (RGB: 148|50|1) or green (RGB: 0|113|0). The two singleton distractor colours were presented equally often but were randomly chosen on each trial. There were two different search display configurations (see Fig. 1B for examples): Either the singleton distractor was presented laterally at one of the two positions on the horizontal midline and the target centrally at one of the two positions on the vertical midline, or the target was presented at a lateral position and the singleton distractor at a central position. Thus, only four of the eight positions in the search display could be occupied by a target or a singleton distractor.

The centrally presented cues subtended 0.8 dva and were diamond shaped, with one side grey (RGB: 134|134|134) and the other side either red or green—the singleton distractor colours—for predictive cues (Fig. 1C) or blue (RGB: 1|96|173) for nonpredictive cues. All colours (grey, red, green and blue) were equiluminant. The reward feedback at the end of the trial (+9, +1, or +0) subtended 1.1 dva. All stimuli were presented against a grey background (RGB: 211|211|211).

### Task, procedure, and design

In a cued additional singleton search task, participants had to report the orientation of the line embedded in the diamond-shaped target while ignoring a salient colour distractor presented among other nontargets. The trial procedure is illustrated in Fig. 1A. Each trial started with the presentation of a central distractor cue for 200 ms. Cues had one coloured side, which pointed to the top, right, bottom, or left side of the display. Figure 1B shows the different cue types. In predictive trials, the cue indicated both the location and the colour (red or green) of the upcoming singleton with 100% validity. Nonpredictive cues were randomly chosen on each trial and neither their colour (blue) nor the location of the coloured side was predictive of the upcoming singleton distractor. Participants were fully informed about the meaning and validity of the cues. They were encouraged to make use of the information provided by predictive cues, which would help them not to get distracted by the salient-but-irrelevant distractor, and to ignore the nonpredictive cues. After an interval of 1,000 ms, the search display was presented for 200 ms. Participants’ task was to report the orientation (horizontal or vertical) of the line embedded in the diamond-shaped target. The target was presented along with seven circle-shaped nontargets, one of which was the singleton that deviated from all other items in its colour: It was either green or red, while all others were grey. Participants responded with a button press, which they were instructed to do as accurately and as quickly as possible. The assignment of response buttons to line orientations was counterbalanced across participants. After the search display, a response display was presented until response or for up to 1,200 ms, leaving participants with a maximal time of 1,400 ms from search onset to respond. If participants failed to respond within this time window, the words “zu langsam” (English: “too slow”) briefly appeared and these trials were later removed from the analyses. After the response, reward feedback was presented for 800 ms. Correct responses were rewarded with either a high (+ 9 points) or a low (+ 1 point) reward. There was no reward (+0 points) for incorrect responses. Reward magnitude was coupled to the colour of the singleton distractor: Either red or green distractors were associated with a low reward, and the other with a high reward. Participants were not informed about this association between distractor colour and reward magnitude. To determine if they were nevertheless aware of this association, we asked them in a postexperimental questionnaire if they thought that reward magnitude was systematically varied and if so, in what way. The reward assignment was counterbalanced across participants but constant for each participant. Figure 1A shows an example of a trial with a predictive cue and a green distractor associated with a high reward. At the end of the experiment, reward points were converted into monetary reward (1€ for 1,250 points). The intertrial interval varied randomly between 700 ms and 1,200 ms (steps of 100 ms).

The experiment consisted of 1,280 trials, organized in 32 blocks of 40 trials each. In the middle of each block of trials and between blocks, participants had the opportunity to take a short break. Trials were equally distributed among the conditions of the 2 × 2 × 2 design: cue condition (predictive vs. nonpredictive), reward (low vs. high) and search display configuration (distractor lateral and target central versus target lateral and distractor central). After the experiment, participants filled in a questionnaire. This questionnaire served to ensure that no inappropriate strategies were used (e.g., ignoring the cues altogether) and to assess if participants were aware of the association between reward magnitude and distractor colour.

### Behavioural analyses

Trials, in which participants failed to respond within the response time limit (on average, 0.5% of all trials) or responded unusually late or early (outlier criterion: ±2.5 *SD* from individual mean reaction time; on average, 2.9% of all trials), and trials that contained ocular or muscle artifacts in the EEG (see below; on average, 3.9% of all trials) were excluded from the analyses. Mean reaction times and accuracy in percentages were computed separately for trials with predictive cues and trials with nonpredictive cues, and for trials with a low-reward distractor and trials with a high-reward distractor. Individual measures were then submitted to repeated-measures analyses of variance (ANOVAs), with the factors cue condition (predictive vs. nonpredictive) and reward (low vs. high). For reaction times—our primary measure of interest—we additionally examined the development of cueing and reward effects over the course of the experiment by splitting the data into four blocks of 320 trials each and running a three-way ANOVA with block (1–4) as the third factor. We also tested cueing and reward effects in each block against zero to determine when these effects emerged; as we predicted effects in a certain direction (see rationale), we used one-tailed *t* tests for these comparisons. When sphericity was violated, the Greenhouse–Geisser epsilon is reported along with the corrected *p* values and the uncorrected degrees of freedom.

### EEG recording and analyses

The EEG was recorded with Ag/AgCl active electrodes (actiCAP, Brain Products, Munich, Germany) from 64 scalp sites, placed according to the International 10–20 system. The vertical (vEOG) and horizontal electrooculogram (hEOG) were computed as the difference between voltage recorded at electrodes positioned above and below (vEOG) and to the left and right (F9/F10) of the eyes. AFz served as the ground electrode. All electrodes were referenced to FCz and rereferenced offline to the average of all electrodes. Impedances were kept below 5 kΩ. The signal was recorded at a sampling rate of 1000 Hz with a high cutoff filter of 250 Hz and a low cutoff filter 0.016 Hz.

EEG preprocessing and analyses were performed in MATLAB (The MathWorks) using the Fieldtrip toolbox (Oostenveld et al., 2011) and custom scripts. The EEG was segmented into epochs of 700 ms, starting 200 ms before the onset of the search display. The 200-ms prestimulus period served as baseline. We excluded trials that were identified as reaction time outliers (±2.5 *SD* from individual mean reaction time), trials with incorrect responses, and trials with ocular artifacts—blinks (vEOG > 80 μV) or eye movements (hEOG > 50 μV)—in the time window of interest (−200 to 350 ms relative to the onset of the search display) from the analyses. Additionally, we removed segments in which the absolute voltage in the channels of interest (PO3/4, PO7/8) exceeded 80 μV.

Contralateral and ipsilateral ERPs were calculated for a parieto-occipital electrode pool (PO3/4, PO7/8), for each participant and separately for cue conditions (predictive vs. nonpredictive), reward conditions (low vs. high), and the two search display configurations (distractor lateral and target central vs. target lateral and distractor central). To determine the time windows for the statistical analyses of the lateralized ERP components of interest, we averaged segments across cue conditions, reward conditions and participants but separately for trials with a lateral distractor and a lateral target. We then calculated difference waveforms by subtracting ipsilateral from contralateral activity and visually inspected for positive or negative deflections in the time range, in which the ERP components of interest are typically observed (see Supplementary Fig. 5 for these grand-averaged differences waves). Time windows were centred on the peak of each deflection (rounded up or down to the closest 5-ms step; e.g., from 243 ms to 245 ms). For trials with lateral targets and central distractors, the N2pc was analysed in the time window from 210 to 310 ms. For trials with lateral distractors and central targets, three lateralized positivities were observed, and mean amplitudes were accordingly computed for three time windows. Based on their timing, we consider these positivities to be a Ppc (105–185 ms; likely reflecting sensory imbalances when singleton distractors are present; see, e.g., Barras & Kerzel, 2016; Fortier-Gauthier et al., 2012; Jannati et al., 2013), an early P_D_ (205–245 ms) and a late P_D_ (290–330 ms).

We show the contra- and ipsilateral waveforms (Fig. 3) but performed all statistical analyses on the difference waveforms (contra- minus ipsilateral activity) to facilitate the interpretation of interaction effects. In a first step, we tested the mean amplitudes of all lateralized ERP components—averaged across conditions—against zero to confirm their overall presence (one-tailed *t* tests, as each component is defined by a certain polarity). In a second step, we ran two-way repeated-measures ANOVAs to test whether the ERP components were modulated by cue condition (predictive vs. nonpredictive) and/or reward (low vs. high).

## Results

### Behavioural results

**Fig. 2:**
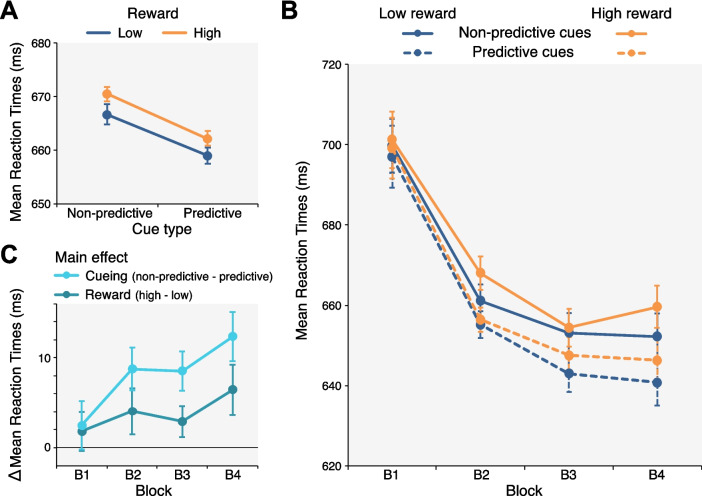
Behavioural results. Mean reaction times shown separately (**A**) for cue conditions (nonpredictive vs. predictive cues) and for trials with a distractor associated with a low or a high reward, and (**B**) for cue and reward conditions across the experiment (divided into four equal blocks of trials). Errors bars represent within-subject standard errors of the means (Cousineau, 2005; Morey, 2008). **C** Cueing and reward effects—calculated as the difference in mean reaction times in trials with nonpredictive versus predictive cues, and trials with a low-reward and a high-reward distractor, respectively—as a function of block. Error bars show standard errors of the means. (Colour figure online)

Overall, mean reactions times were faster with predictive cues (661 ms ± 12 ms; Mean ± *SEM*) than with nonpredictive cues (669 ms ± 12 ms), revealing a numerically small but significant cueing benefit, *F*_(1,49)_ = 15.65, *p* <.001, partial η^2^ =.242 (Fig. 2A; individual means and their distribution are shown in Supplementary Fig. 1). The reward manipulation likewise affected reaction times, *F*_(1,49)_ = 6.93, *p* =.011, partial η^2^ =.124: Responses were slower in trials with a high-reward distractor (666 ms ± 12 ms) than in trials with a low-reward distractor (663 ms ± 12 ms). As is evident in Fig. 2A, the effects of cueing and reward did not interact but influenced reaction times in an independent, additive manner, *F*_(1,49_) =.02, *p* =.884.

To examine the emergence of cueing and reward effects across the experiment, we divided trials into four equal blocks of trials and included block as an additional factor. Figure 2B shows reaction times as a function of block separately for nonpredictive (solid lines) and predictive (dashed lines) and for low-reward (blue/dark grey) and high-reward distractors (orange/light grey). The three-way ANOVA confirmed the main effects of cue condition, *F*_(1,49)_ = 16.32, *p* <.001, partial η^2^ =.250, and reward, *F*_(1,49)_ = 6.82, *p* =.012, partial η^2^ = 122, as well as the absence of an interaction of these two factors, *F*_(1,49)_ = 0.16, *p* =.693. As typically observed in visual search tasks, reaction times generally decreased over the course of the experiment, as evidenced by an effect of block, *F*_(3,147)_ = 17.52, *p* <.001, partial η^2^ =.263, ε =.576. More importantly, an interaction between block and cue condition, *F*_(3,147)_ = 2.95, *p* =.035, partial η^2^ =.057, revealed that cueing benefits—calculated as the difference in reaction times with nonpredictive and predictive cues—increased over the course of the experiment (Fig. 2C), only emerging in the second block of trials, *B1*: *t*_(49)_ = 0.92, *p* =.182; *B2*: *t*_(49)_ = 3.07, *p* =.002, *d* =.43; *B3*: *t*_(49)_ = 3.35, *p* <.001, *d* =.474; *B4*: *t*_(49)_ = 3.74, *p* <.001, *d* =.53; one-tailed *t* tests against zero. Similarly, reward effects—calculated as the difference in reaction times with high- and low-reward distractors—emerged during the second block of trials (Fig. 2C), *B1*: *t*_(49)_ = 0.81, *p* =.210; *B2*: *t*_(49)_ = 1.71, *p* =.047, *d* =.24; *B3*: *t*_(49)_ = 1.49, *p* =.071; *B4*: *t*_(49)_ = 2.23, *p* =.015, *d* =.32, but they remained relatively constant thereafter, *B1* vs. *B4*: *t*_(49)_ = 1.36, *p* =.179. The interaction between the factors block and reward was not significant, *F*_(3,147)_ = 0.83, *p* =.463, ε =.843. There was also no three-way interaction, *F*_(3,147)_ = 0.82, *p* =.486. Thus, neither the overall decrease of reaction times nor the increase of cueing benefits over the course of the experiment depended on the magnitude of the reward associated with the singleton distractor.

To determine whether awareness of the association between singleton distractor colour and reward magnitude modulated the influence of reward on search performance, we additionally analysed reaction times separately for participants who described the correct colour–reward association in the postexperimental questionnaire (*n* = 16) and participants who reported that they were unaware of any systematic variation of reward magnitude (*n* = 28). Six participants believed that reward was systematically varied but had the wrong idea about how—for example, they described that it depended on reaction times or where stimuli were presented; we did not include these participants in the analyses. Reaction times were slower in trials with a high-reward distractor than in trials with a low-reward distractor in both participants who were aware of the reward contingency, *F*_(1,15)_ = 9.39, *p* =.008, partial η^2^ =.385, and in participants who were not aware, *F*_(1,27)_ = 5.12, *p* =.032, partial η^2^ =.159. The effect was somewhat more pronounced in aware participants than in unaware participants (7.15 ms ± 2.33 ms vs. 4.11 ms ± 1.82 ms; note that this is the overall effect across all trials—effects towards the end of the experiment were much larger; see Fig. 2B–C), but when included as between-subject factor, awareness did not interact with reward condition, *F*_(1,42)_ = 1.04, *p* =.314. These findings are in line with several previous studies demonstrating that reward can influence the allocation of attention via implicit learning mechanisms, albeit effects tend to be larger in participants aware of reward contingencies (e.g., Anderson, 2015; Anderson et al., 2021; Bourgeois et al., 2017; Le Pelley et al., 2015). Curiously, cueing benefits—which were present in both groups, *aware* : *F*_(1,15)_ = 16.45, *p* =.001, partial η^2^ =.523; *unaware: F*_(1,27)_ = 7.94, *p* =.009, partial η^2^ =.227—were also more pronounced in aware participants (14.75 ms ± 3.64 ms) than in unaware participants (5.87 ms ± 2.09 ms); as a between-subject factor, awareness significantly interacted with cue condition, *F*_(1,42)_ = 5.22, *p* =.028, partial η^2^ =.110. Cue condition and reward magnitude did not interact in either group of participants, *aware: F*_(1,15)_ = 3.11, *p* =.098, partial η^2^ =.172; *unaware: F*_(1,27)_ = 2.55, *p* =.122, partial η^2^ =.086.

To confirm that participants ignored the nonpredictive cues as instructed, we also analysed mean reaction times in the nonpredictive cueing condition separately for trials, in which the target or the singleton distractor appeared at the “cued” location, relative to when one of the two remaining items appeared at that location. As any automatic shifts of attention following nonpredictive cues were more likely to occur at the beginning of the experiment, we did this separately for each of the four blocks of trials (Supplementary Fig. 3). While there was overall a slight tendency, at least numerically and in some blocks, for responses to be delayed when the target position was nonpredictively cued, and sped up when the distractor position was “cued”—a pattern that would indicate that the nonpredictively cued locations might have been suppressed to some degree—none of these differences were significant except for one: In Block 2, responses were slower when the target appeared at the nonpredictively cued location, *t*_(49)_ = 3.04, *p* =.004, *d* =.43. Thus, participants were mostly able to ignore the nonpredictive cues.

Search accuracy was overall very high (93.25% ± 0.67%) and did not differ between reward conditions (*low*: 93.37% ± 0.67%; *high*: 93.13% ± 0.67%), *F*_(1,49)_ = 2.00, *p* =.164, or cue conditions, (*nonpredictive*: 93.19% ± 0.69%; *predictive*: 93.30% ± 0.66%), *F*_(1,49)_ = 0.26, *p* = 0.614. Cueing and reward effects did not interact, *F*_(1,49)_ = 0.92, *p* =.976.

### Electrophysiological results

**Fig. 3:**
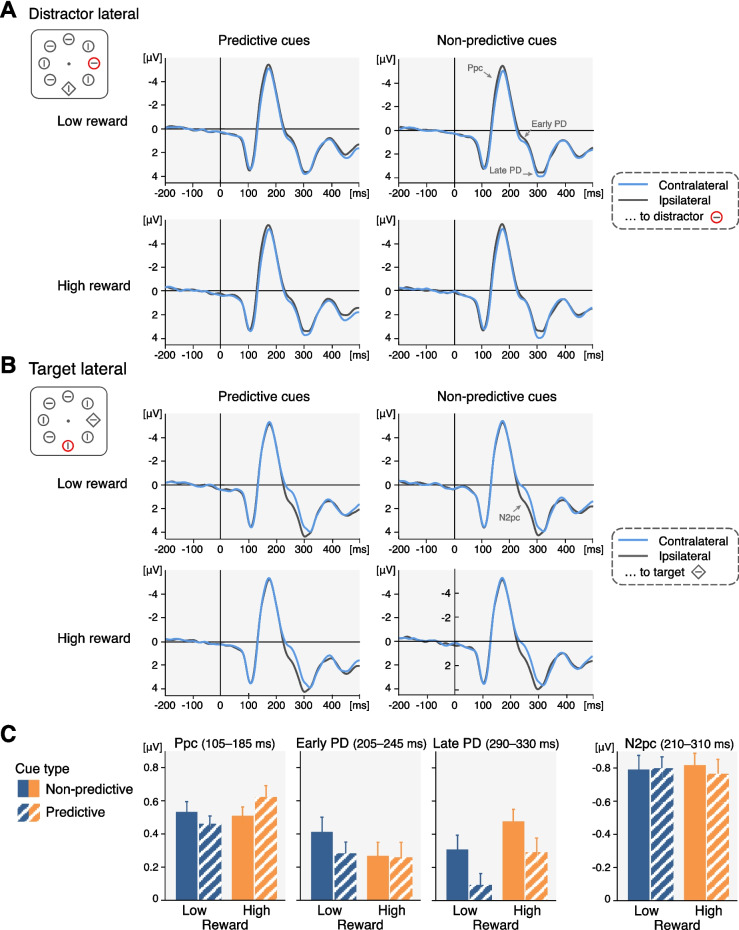
Electrophysiological results. Grand-averaged contralateral and ipsilateral waveforms at parieto-occipital electrodes (PO3/4, PO7/8) in trials with (**A**) a lateral singleton distractor and a central target and (**B**) a lateral target and a central singleton distractor. ERP waveforms are time-locked to the onset of the search display and shown separately for cue types (columns) and reward magnitudes (rows). For illustration purposes, the waveforms were lowpass filtered at 35 Hz. **C** Mean amplitudes of all ERP components of interest (Ppc, early PD and late PD elicited by lateral distractors and NT elicited by lateral targets) in the different cueing and reward conditions. Errors bars represent within-subject standard errors of the means (Cousineau, 2005; Morey, 2008). (Colour figure online)

In trials with a lateral distractor and a central target, we analysed the Ppc (105–185 ms), the early P_D_ (205–245 ms) and the late P_D_ (290–330 ms). Contralateral and ipsilateral waveforms elicited by lateral distractors are shown in Fig. 3A, separately for low- and high-reward distractors and for trials with predictive and nonpredictive cues (the corresponding difference waves are shown in Supplementary Fig. 4). The mean amplitudes of all ERP components are plotted in Fig. 3C (individual mean P_D_ amplitudes and their distribution are shown in Supplementary Fig. 2).

The overall presence of a Ppc was statistically confirmed, t_(49)_ = 8.30, *p* <.001, *d* = 1.17; one-tailed *t* test against zero, but its mean amplitude was not modulated by cue condition, *F*_(1,49)_ = 0.10, *p* =.760, or reward condition, *F*_(1,49)_ = 1.23, *p* =.272. The factors of cue type and reward did not interact, *F*_(1,49)_ = 3.02, *p* =.088.

We obtained similar results for the early P_D_: It was present, *t*_(49)_ = 4.07, *p* <.001, *d* = 0.58, but it did not differ between cue, *F*_(1,49__)_ = 0.77, *p* =.384, or reward conditions, *F*_(1,49)_ = 0.71, *p* =.403, and neither was there an interaction of the two factors, *F*_(1,49)_ = 0.81, *p* =.373.

The late P_D_, *t*_(49)_ = 3.50, *p* <.001, d =.50, by contrast, was modulated by both cue type, *F*_(1,49)_ = 6.30, *p* =.015, partial η^2^ =.114, and reward, *F*_(1,49)_ = 4.77, *p* =.034, partial η^2^ =.089. It was larger (i) for distractors associated with a high reward (0.38 µV ± 0.10 µV) than for distractors associated with a low reward (0.20 µV ± 0.09 µV) and (ii) for distractors following nonpredictive cues (0.39 µV ± 0.09 µV) than for distractors following predictive cues (0.19 µV ± 0.09 µV). The effects of reward and cue type were roughly additive and did not interact, *F*_(1,49)_ = 0.04, *p* =.849.

In trials with a lateral target and a central distractor, we analysed the target N2pc (210–310 ms). The contralateral and ipsilateral waveforms elicited by lateral targets following nonpredictive or predictive cues in trials with a low- or a high-reward distractor are shown in Fig. 3B. We observed the same pattern of results as for the Ppc and the early P_D_: A target N2pc was present, *t*_(49)_ = −7.50, *p* <.001, *d* = −1.06, but not affected by manipulations of cue type, *F*_(1,49)_ = 0.01, *p* =.940, the magnitude of reward associated with the distractor, *F*_(1,49)_ = 0.13, *p* =.719, or a combination of cue type and reward, *F*_(1,49)_ = 0.37, *p* =.548.

## Discussion

With this study, we set out to test the efficacy of precueing salient distractors under conditions that should provide a high incentive to make use of this information by anticipatorily suppressing the respective distractor features: Cues were spatially specific, fully valid and for correct responses, participants received points that translated to monetary reward. We show that distractors can be actively suppressed in an anticipatory manner, at least under such “optimal” conditions: Responses were faster when the search display was preceded by predictive cues indicating the location and identity of the colour singleton distractors relative to cues that were nonpredictive. These cueing benefits increased over the course of the experiment, likely due to increasing practice with the cues and how to implement suppression. Experiencing that making use of the cues was helpful for performing the task might also have boosted the incentive to suppress even further over time. Electrophysiological evidence complemented this pattern of task performance: The amplitude of the late P_D_, an established marker of active suppression (e.g., Feldmann-Wüstefeld & Vogel, 2018; Feldmann-Wüstefeld et al., 2020; Gaspelin & Luck, 2018b, c), was smaller for distractors following predictive cues than for distractors following nonpredictive cues, indicating that less suppression was required upon distractor presentation when their location and colour was known in advance (see also Heuer & Schubö, 2019). Importantly, the target N2pc—which reflects target enhancement (e.g., Hickey et al., 2009; Munneke et al., 2013)—was not modulated by cue type, confirming that the cues selectively affected distractor processing: They were used to implement suppression rather than to facilitate processing at the remaining potential target locations.

Together, these convergent behavioural and electrophysiological findings demonstrate that knowledge about upcoming salient distractors *can* be exploited to facilitate their suppression (see also Chang et al., 2018; Heuer & Schubö, 2019; Van Zoest et al., 2021). But that is not always the case: As there are also a number of previous studies that failed to observe benefits of precueing the features of salient or nonsalient distractors (e.g., Beck & Hollingworth, 2015; Becker et al., 2015; Salahub & Emrich, 2021; Wang & Theeuwes, 2018a), future research should focus on identifying the boundary conditions under which successful suppression can be achieved. Assuming that anticipatory top-down suppression is an effortful operation, we propose that motivation is one key ingredient that determines whether participants attempt to use the cues to prepare for upcoming distraction. If, however, the incentive to suppress is indeed critical, matters are complicated by the fact that incentive will likely be influenced by many different factors, ranging from experimental design choices (e.g., cue validity and specificity, task difficulty or timing) to state and trait characteristics of the sample (e.g., arousal, fatigue, achievement motivation, conscientiousness). These factors can be expected to interact, making it virtually impossible to pinpoint how exactly one particular factor independently affects the efficacy of distractor cueing and thus suppression. A cue validity of 70%, for example, might provide enough incentive in a very difficult task, when errors are likely to occur anyway and the potential benefit is much greater than the potential cost, but not in an easy task. It is thus important to take task context and individual differences into consideration before drawing firm conclusions about the potency of top-down control when it comes to preparing for distraction.

The overall cueing benefits we observed were relatively small; on average, responses following predictive cues were only 8 ms faster than responses following nonpredictive cues. One might thus wonder if voluntary suppression can truly confer a behavioural advantage worth mentioning. However, we believe that this average benefit hardly represents the upper bound of what distractor suppression based on advance knowledge can achieve. First, this type of suppressions seems to become more effective with more practice: Cueing benefits only emerged in the second block of trials, and still increased considerably even in the last block of trials (up to 12 ms). It is thus conceivable that more practice would yield larger benefits. In this regard, it is important to keep in mind that practice with voluntary suppression cannot be equated with experience-dependent suppression based on implicit learning in this study—predictive cues provided information on a trial-to-trial basis that could not be inferred from statistical learning of distractor regularities. Second, there are considerable interindividual differences in the benefits brought about by voluntary suppression (see also Heuer & Schubö, 2019). For instance, the largest individual cueing benefit towards the end of the experiment (i.e., in Block 4) was 79 ms, but, at the other end of the distribution, there was also a participant with a cueing “cost” of 52 ms. While one could argue why individual cueing effects differ that much (e.g., motivation, fatigue, attentional control abilities etc.), they certainly showcase that this form of suppression can yield substantial behavioural advantages. Third, even though we aimed to promote the efficacy of suppression, we did not take all thinkable measures to create “optimal” conditions and there are still aspects of the task that might have discouraged suppression. For example, we assume that a task has to be fairly difficult, at least in part due to distracting information, so that participants are actually motivated to make use of cues that could help reduce that distraction and thereby improve performance. However, for practical reasons, we chose not to include trials without a distractor and used the nonpredictive condition as a baseline instead, even though capture by the distractor is typically reduced when distractor probability is high and distractors are present on consecutive trials. The fact that performance nonetheless improved with predictive cues shows that distractors were still disrupting visual search, but behavioural improvements might be larger if the distractor’s potential to capture attention is higher.

Suppression mechanisms can operate at different points in time and are thus often characterized by their timing: Whereas proactive suppression occurs before a distractor appears (or before the first shift of attention), reactive suppression operates after distractor onset or even after initial attentional capture (Liesefeld et al., 2024). We would like to clarify that we use the term “active” primarily to distinguish the form of suppression studied here from passively not attending to something on the one hand, and from involuntary, experience-dependent suppression that emerges from implicit learning on the other hand. It is not meant to imply a proactive suppression mechanism; we remain largely agnostic with respect to the timing of suppression in this study and refrain from drawing any strong conclusions. However, a few aspects of the findings clearly relate to this matter and invite speculation. The modulation of the P_D_ occurred relatively late and well after the onset of the target N2pc, thus indicating a reactive process after the initial shift of attention towards the target. This reactive suppression might prevent subsequent distraction or facilitate disengagement from the distractor—both can be assumed to promote target processing and could account for behavioral improvements. Our findings and these considerations do not preclude the possibility, though, that proactive processes were involved (albeit our analyses did not reveal any direct electrophysiological evidence). Predictive cues provided information about the upcoming distractors long before their onset, and it is conceivable that this advance knowledge might have induced something like a heightened state of readiness to suppress. Such a state could mean that suppression after eventual distractor appearance was more efficient, manifesting in smaller late P_D_ amplitudes. The absence of an N2pc towards the distractor could also be interpreted as evidence in favour of a proactive nature of suppression.

There are alternative accounts that challenge the idea that there even is such a thing as “suppression”, arguing that most findings in favour of attentional suppression could be explained by other processes such as passive ignoring or attending somewhere else (e.g., Kerzel et al., 2021). In our paradigm, for example, one could argue that, in predictive trials, attention is simply shifted towards the hemifield opposite the cued side. This could result in the same event-related potential: A negativity contralateral to the one hemifield would just be the flipside of a positivity contralateral to the other; these two possibilities are methodologically indistinguishable. While this idea has been directly challenged (e.g., Drisdelle & Eimer, 2021) and we believe it to be a particularly unlikely scenario in this study (e.g., the side opposite the cued side never contained a target), we cannot positively rule it out based on our findings. However, this seems to be, to a large extent, a question of terminology and how exactly one defines “suppression” (see also Liesefeld et al., 2024). Our main conclusion—that it is possible to prepare for and reduce distraction by a salient stimulus on a trial-to-trial basis if the incentive to do so is high—is not affected by these considerations. In other words, the functionality and outcome of the process would remain the same if we used a different label.

To increase participants’ motivation to use the distractor cues in this task, we did not only reward them for correct responses but also varied reward magnitude as a function of distractor colour, reasoning that a higher reward would provide a higher incentive to suppress than a low reward (see also Gong et al., 2016). In line with previous findings (Anderson et al., 2013; Failing & Theeuwes, 2015; Feldmann-Wüstefeld et al., 2016; Le Pelley et al., 2015), reaction times were longer when high-reward distractors were present, indicating that these interfered more with target selection than low-reward distractors. Contrary to our expectations, however, this was the case for both predictive as well as nonpredictive cues; that is, cue type and reward affected search performance independently. Again, this behavioural performance pattern was mirrored in the P_D_, which was larger for high-reward distractors than for low-reward distractors irrespective of cue condition, reflecting the increased need for suppression. Different explanations for this lack of a differential effect of reward magnitude are conceivable. For one, the difference between low and high reward might have been too small to differentially modulate distractor suppression. Given that similar reward schemes have been shown to affect attentional mechanisms quite substantially (e.g., Heuer & Schubö, 2018; Heuer et al., 2017; Hickey et al., 2010; Theeuwes & Belopolsky, 2012), this seems to be an unlikely explanation, though. Second, along similar lines, the slightly increased incentive to suppress might not have been powerful enough to overcome or reduce the interference caused by high-reward distractors. Indeed, in the study by Gong et al. (2016), who found suppression to be more effective when the distractor was associated with a high-reward as compared with a low-reward condition, distractors did not differ in physical salience. While a high reward might boost the suppression of nonsalient distractors, distractors that are salient with respect to both their physical properties as well as their value are likely particularly difficult to suppress (see also Gong & Liu, 2018). Third, it is possible that the general prospect of reward globally enhances motivation, but that reward magnitude does not differentially affect how successfully reward-associated stimuli can be suppressed. According to this line of reasoning, the incentive to suppress would influence the efficacy of distractor suppression in an all-or-nothing fashion: Participants are either motivated enough to use the cues or not. Once they try, their ability to make use of that advance information for distractor suppression does not scale with their exact level of motivation, but depends on other factors (e.g., cognitive control). So even if different reward magnitudes did induce slight differences in the incentive to suppress low- versus high-reward distractors, these would not manifest in behaviour. Finally, it is conceivable that reward was not anticipated at all in this task but only took effect upon search onset—that is, only when reward-associated information was present did it make a difference if a high or a low reward was at stake. That way, reward could affect search performance but not interact with the information about the upcoming distractor provided by the cues and the resulting behavioural benefit.

As we leveraged several tools to maximize the incentive to suppress without manipulating them independently—most notably cue specificity, cue validity and reward—our design does not enable us to isolate the specific contribution of each of them. This has several implications for the conclusions that can be drawn based on our results. For example, one might wonder if reward contributed to increasing the incentive to suppress at all, given that a higher reward even impaired search performance. A finding that can be taken as evidence that reward did, in fact, increase motivation is that participants who became aware of the association between distractor colour and reward not only exhibited larger effects of reward but also larger cueing benefits. However, the causal direction here cannot be established: It is also possible that participants who were just generally more motivated and invested in the task from the start suppressed more successfully and were more likely to figure out the reward scheme. To determine whether the presence of reward by itself increases the incentive to prepare for upcoming distraction, measurably improving performance, a baseline condition without reward will be necessary.

Similarly, we cannot draw any conclusions about the relative contributions of spatial and feature-based suppression—an issue that is of considerable theoretical interest, as there is some controversy about whether feature-based suppression is possible or not (e.g., Gaspelin & Luck, 2018b; Luck et al., 2021; Theeuwes et al., 2022). However, the cues in our task always provided valid information about either both distractor location and colour, or neither. Thus, even if reward (associated with colour) interacted with the benefit conferred by predictive cues, this would not necessarily imply feature-based suppression, as this effect could easily be mediated by the simultaneously cued location.

This study provides proof-of-principle that trial-to-trial advance knowledge can be used to effectively suppress salient distractors, yielding measurable performance benefits. Taken together with previous studies that obtained mixed evidence on the efficacy of precueing distractors, our findings highlight the importance of considering the voluntary and effortful nature of top-down suppression and thus its dependence on motivation—and consequently its dependence on all experimental, situation and person variables that shape participants’ incentive to suppress.

## Supplementary Information

Below is the link to the electronic supplementary material.

Supplementary file1 (PDF 239 KB)

## Data Availability

The data are available at the Open Science Framework (https://osf.io/hp4m7/).

## Footnotes

1 Note that we set out to create optimal conditions for the suppression of distracting information based on advance knowledge, not optimal conditions for participants not to get distracted. Therefore, we did not aim to render the singleton distractor easier to ignore by any means other than the information provided by the cues (e.g., by encouraging feature-search mode; Gaspelin et al., 2015; Gaspelin & Luck, 2018b). To the contrary, we reasoned that distractors that were likely to capture attention would incentivize participants to make use of available information that might help them to suppress these distractors.

## Availability of code

Code will be made available upon request.
