# Supplementary material for: Cueing distractors is effective when the incentive to suppress is high
Source: Atten Percept Psychophys. 2025 May 5;87(5):1416–30. doi: 10.3758/s13414-025-03075-w (PMC12204925; doi:10.3758/s13414-025-03075-w)
Supplement: Supplementary file 1 — Supplementary file1 (PDF 239 KB) [file 13414_2025_3075_MOESM1_ESM.pdf]

Supplementary Material

Cueing distractors is effective when the incentive to suppress is high

Anna Heuer and Anna Schubö

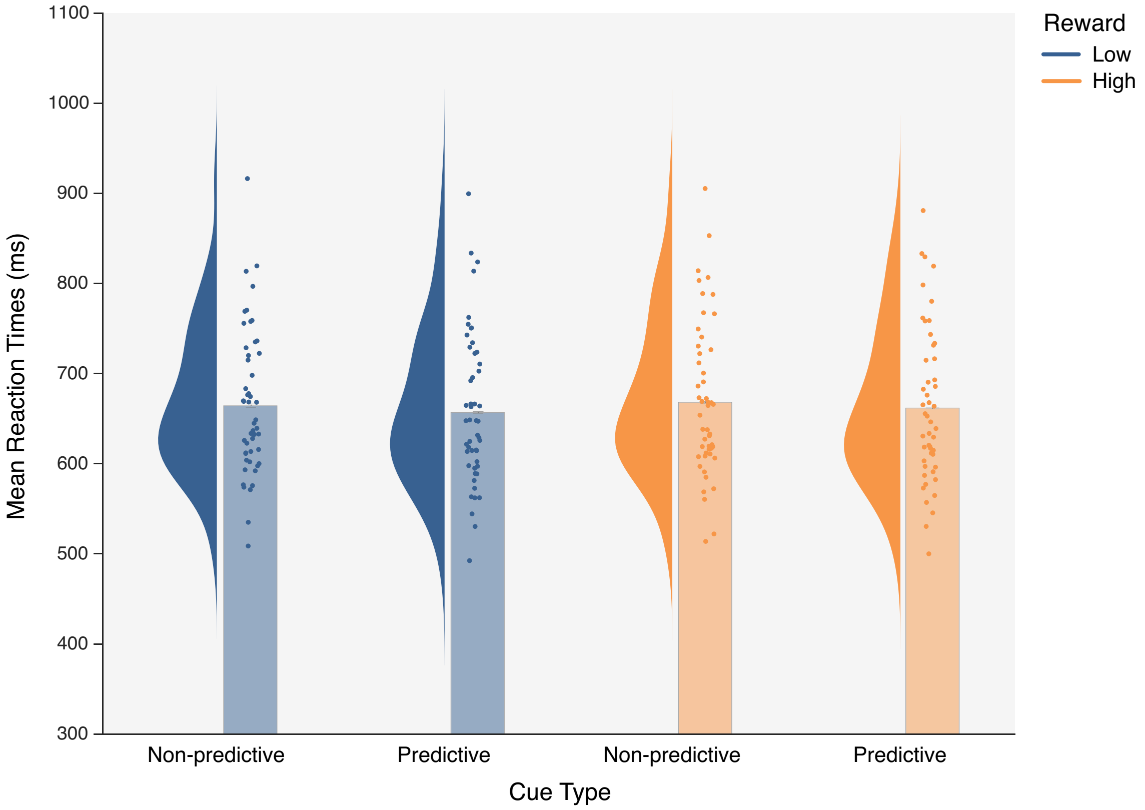

Supplementary Fig. 1. Mean reaction times shown separately for cue type (non-predictive vs. predictive) and reward conditions (low vs. high). Raincloud plots (modified from Allen, Poggiali, Whitaker, Marshall, & Kievit, 2019) show the individual means, their distribution (probability density function) and the group mean for each condition. Error bars represent within-subject standard errors of the means (Cousineau, 2005; Morey, 2008).

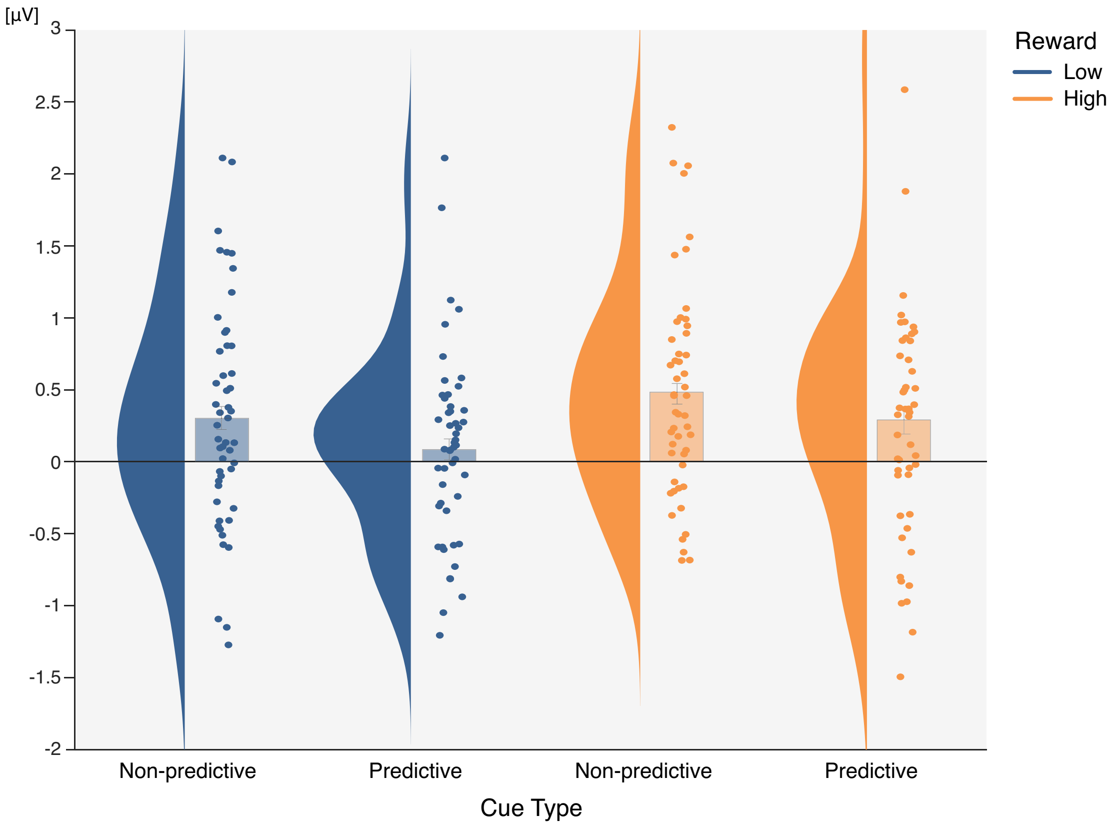

Supplementary Fig. 2. Mean amplitudes of the PD shown separately for cue type (non-predictive vs. predictive) and reward conditions (low vs. high). Raincloud plots (modified from Allen et al., 2019) show the individual means, their distribution (probability density function) and the group mean for each condition. Error bars represent within-subject standard errors of the means (Cousineau, 2005; Morey, 2008).

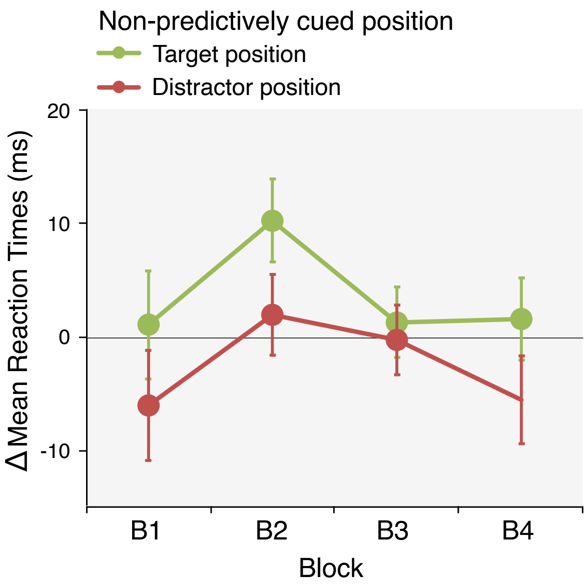

Supplementary Fig. 3. Mean reaction times shown separately for trials, in which the non-predictive cue pointed towards the target position or the distractor position, relative to reaction times when the non-predictive cue pointed towards one of the remaining two locations. Accordingly, positive values indicate that responses were slower than in trials, in which the cue pointed towards one of two locations with neither target nor distractor; negative values indicate the opposite pattern.

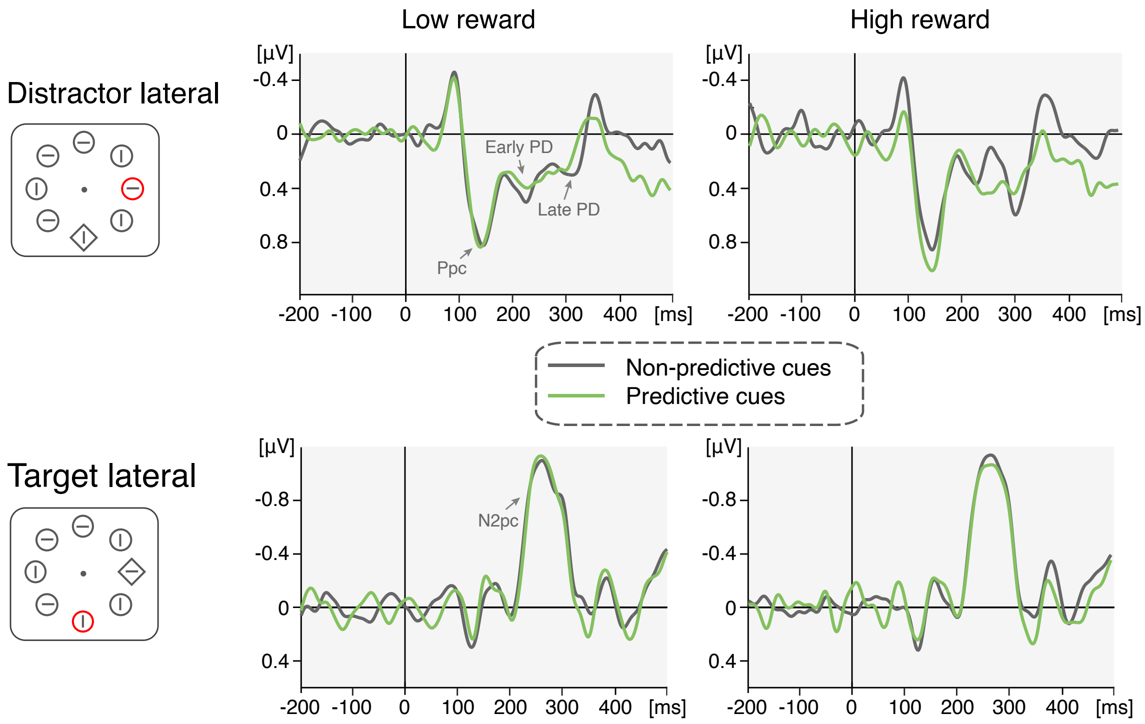

Supplementary Fig. 4. Difference waves (contralateral minus ipsilateral activity; see. Fig. 3) of grand-averaged ERPs at parieto-occipital electrodes (PO3/4, PO7/8) time-locked to the onset of the search display, shown separately for search display configurations, reward magnitudes and cue types. For illustration purposes, the waveforms were lowpass filtered at 35 Hz.

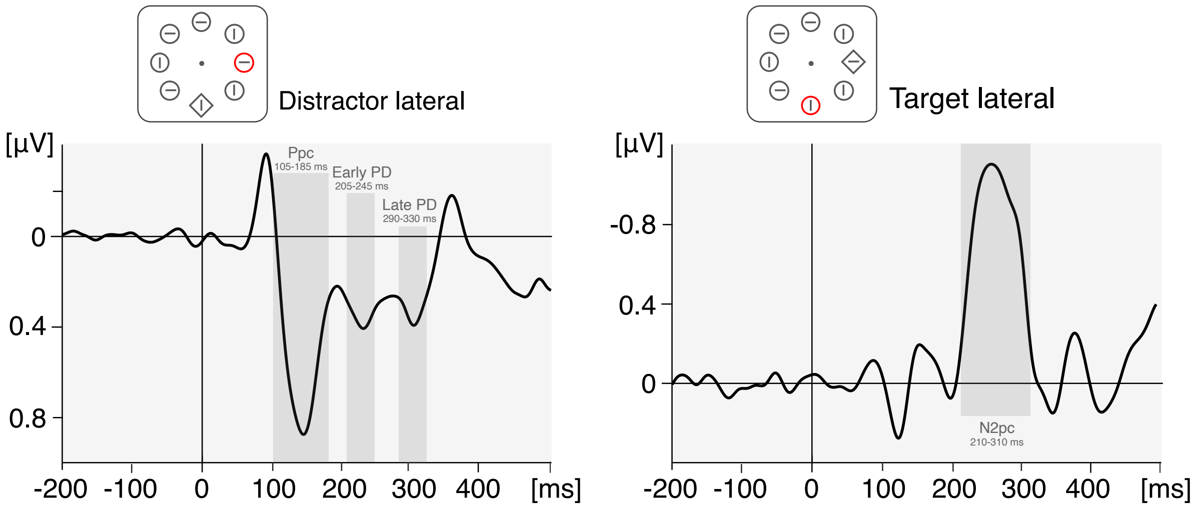

Supplementary Fig. 5. Difference waves (contralateral minus ipsilateral activity; see. Fig. 3) of grand-averaged ERPs at parieto-occipital electrodes (PO3/4, PO7/8) time-locked to the onset of the search display, collapsed across reward and cue conditions. Based on these difference waves, the time windows of analysis for the ERP components were chosen. For illustration purposes, the waveforms were lowpass filtered at 35 Hz.

References

Allen, M., Poggiali, D., Whitaker, K., Marshall, T. R., & Kievit, R. A. (2019). Raincloud plots: A multi-platform tool for robust data visualization. *Wellcome Open Research*, *4*, 1–51.

Cousineau, D. (2005). Confidence intervals in within-subject designs: A simpler solution to Loftus and Masson's method. *Tutorials in Quantitative Methods for Psychology*, *1*, 42–45.

Morey, R. D. (2008). Confidence intervals from normalized data: A correction to Cousineau (2005). *Tutorials in Quantitative Methods for Psychology*, *4*(2), 61–64.
